# Advances of Antisense Oligonucleotide Technology in the Treatment of Hereditary Neurodegenerative Diseases

**DOI:** 10.1155/2021/6678422

**Published:** 2021-06-10

**Authors:** Mengsi Lin, Xinyi Hu, Shiyi Chang, Yan Chang, Wenjun Bian, Ruikun Hu, Jing Wang, Qingwen Zhu, Jiaying Qiu

**Affiliations:** ^1^Department of Prenatal Screening and Diagnosis Center, Affiliated Maternity and Child Health Care Hospital of Nantong University, Nantong, Jiangsu Province 226001, China; ^2^School of Medicine, Nantong University, Nantong, Jiangsu Province 226001, China; ^3^School of Life Sciences, Nantong University, Nantong, Jiangsu Province 226001, China

## Abstract

Antisense nucleic acids are single-stranded oligonucleotides that have been specially chemically modified, which can bind to RNA expressed by target genes through base complementary pairing and affect protein synthesis at the level of posttranscriptional processing or protein translation. In recent years, the application of antisense nucleic acid technology in the treatment of neuromuscular diseases has made remarkable progress. In 2016, the US FDA approved two antisense nucleic acid drugs for the treatment of Duchenne muscular dystrophy (DMD) and spinal muscular atrophy (SMA), and the development to treat other neurodegenerative diseases has also entered the clinical stage. Therefore, ASO represents a treatment with great potential. The article will summarize ASO therapies in terms of mechanism of action, chemical modification, and administration methods and analyze their role in several common neurodegenerative diseases, such as SMA, DMD, and amyotrophic lateral sclerosis (ALS). This article systematically summarizes the great potential of antisense nucleic acid technology in the treatment of hereditary neurodegenerative diseases.

## 1. Introduction

Neurodegenerative diseases are a range of conditions that are characterized by the progressive functional and structural degeneration of the central or peripheral nervous system, and the patient's cognitive ability and athletic ability are severely impaired, placing a huge burden on the individual's family and society [1]. Neurodegenerative diseases comprise a set of over 600 diseases, including Alzheimer's disease (AD), Parkinson's disease (PD), Huntington's disease (HD), ALS, and multiple sclerosis (MS) [2–4]. Several biological characteristics, such as mitochondrial dysfunction, oxidative stress, abnormal protein accumulation, trophic factor deficiency, and inflammatory response, are accompanied by these diseases, but the pathogenesis is still too complex and diverse to be fully elucidated. Although scientists have made great progress in understanding the environmental and genetic causes of these diseases in the past few decades, the drugs for treating these diseases have so far been limited, and none of them can prevent or mitigate neurodegenerative diseases [5–10].

Antisense oligonucleotides (ASOs) are single-stranded oligonucleotides of 8 to 50 bases in length that specifically bind to selected RNA sequences and regulate the expression of genes by several mechanisms depending on their chemical properties and targets, which will affect protein synthesis at posttranscriptional processing or protein translation levels [11]. In 1978, Zamecnik and Stephenson reported for the first time that chemically synthesized ASOs can inhibit the production of Rous sarcoma virus in chicken embryo fibroblasts in a sequence-specific manner [12]. With the joint efforts of several generations of scientists, the mechanism of action of ASOs has been gradually elucidated, and various chemical modifications have been applied to improve the stability of ASOs. In 1998, the first antisense drug Vitravene (Fomivirsen) was approved by the US FDA [13]. In recent years, the use of antisense oligonucleotide technology for treating neuromuscular diseases has made remarkable progress. In 2016, two antisense nucleic acid drugs were approved by the US FDA for the treatment of neuromuscular diseases, namely, eteplirsen (Exondys 51) for the treatment of DMD and nusinersen (Spinraza) for the treatment of SMA. If ASOs are used to treat neurodegenerative diseases, they must be stable in the body and enter the central nervous system cells to specifically target the causative genes. In this review, we will mainly discuss the development of antisense nucleic acid drugs in three neurodegenerative diseases—SMA, DMD, and ALS.

## 2. Regulatory Mechanisms of ASOs

Antisense oligonucleotides can regulate gene expression by recruiting RNase H to degrade target RNA or prevent splicing factors from binding through steric hindrance to treat neurodegenerative diseases.

### 2.1. RNase H-Mediated RNA Degradation

**Figure 1:**
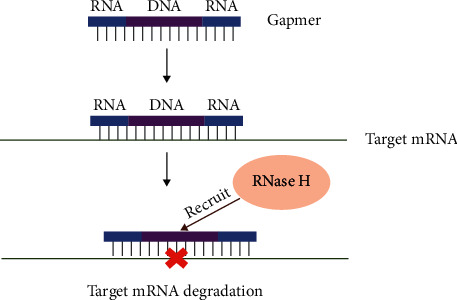
ASOs recruit RNase H to degrade target RNA.

It has been found that the DNA-RNA hybrid is a substrate of the RNase H enzyme, while the main function of RNase H is to degrade RNA-DNA hybrids synthesized by lagging strands in the nucleus. Meanwhile, RNase H is also present in the cytoplasm and can degrade mature mRNA [14, 15]. Oligonucleotides containing DNA bases can induce cleavage of mRNA after bounding to mRNA. Gapmer ASO was designed with two RNA arms modified with resisting nucleases and enhancing affinity of complementary sequences located on either side of the central 8–10 base DNA “gap,” which serves as a substrate for RNase H to induce targeted cleavage of mRNA and results in protein translation inhibition (Figure 1) [16–18]. The pathological feature of many neurodegenerative diseases is the accumulation of toxic proteins, in which many are caused by genetic mutations. If the target protein undertakes important cellular functions, therefore, degradation of the protein is harmful to the cell. In this case, it is necessary to specifically degrade harmful mRNA-containing mutations. Using the principle of base complementary pairing, selective gapmers can be designed to specifically target mRNA with mutation sites to initiate RNase H-mediated degradation [19, 20].

### 2.2. Splicing Regulation

Splicing is a process of removing introns from the initial DNA transcription product and joining the exons to form a contiguous RNA molecule. The eukaryotic mRNA precursor is spliced in the nucleus to produce mature mRNA, which is transported to the cytoplasm and translated into protein. Alternative splicing can produce protein variants with different functions and can produce beneficial proteins or reduce harmful proteins with splicing regulation. Splicing is a complex process regulated by multiple mechanisms involving multiple proteins, such as the mRNA precursor with splicing signals and some splicing factors bind to these signal sequences to perform specific functions. The mRNA precursor has basic splicing signals such as 5′ splice site, 3′ splice site, branch point sequence, and polypyrimidine sequence, as well as regulatory signals such as silencer and enhancer [21, 22].

**Figure 2:**
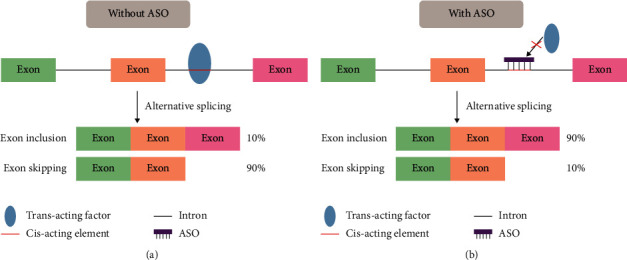
ASO prevents splicing factor binding through steric hindrance.

ASO regulates splicing by binding to the splicing signal on mRNA precursor through base complementary pairing, thereby blocking the binding of splicing factors, which will change the original splicing pattern or lead to the activation of a new splicing site and significant changes in the inclusion of exons and ultimately obtain the desired protein product (Figure 2) [23]. This type of ASO is also called splicing switching oligonucleotides (SSOs). For neurodegenerative diseases, SSO has many potential applications. For example, SSO can reduce harmful isoforms, skip abnormal exons to restore normal transcription, and remove pathogenic mutations from genes or restore the reading frame by removing exons with mutations [24–27]. Both nusinersen and eteplirsen approved by the FDA are involved in regulating splicing.

## 3. Chemical Modification of Antisense Nucleic Acid

The rapid degradation of antisense oligonucleotides by endo- and exonuclease is one of the main factors affecting the effectiveness of antisense oligonucleotides. 3′-5′ exonuclease can degrade serum unprotected antisense oligonucleotides in half an hour [28]. Increasing the dose of ASO will increase off-target effects, increase toxicity, and cause immune responses, which will seriously affect the uptake of ASO by cells [29]. In order to enhance the antisense nucleic acid's ability to resist enzymatic hydrolysis and cellular uptake and, furthermore, improve its stability, researchers have carried out a variety of structural modifications and designs on antisense nucleic acids. There are three main types of chemical modification: backbone modification, glycosyl modification, and other modified ASO.

### 3.1. Backbone Modification

The first-generation oligonucleotide modification refers to the phosphorothioate (P = S) backbone, which is achieved by replacing the nonbridging oxygen atoms in the phosphate group of the nucleotide with a sulfur atom. The addition of sulfur atoms increases the backbone negative density of the antisense nucleic acid, which can enhance the absorption efficiency of ASO by various types of cells [30]. In addition, P = S modified antisense oligonucleotides increase the ability to bind serum proteins, reduce the metabolism of ASO by the kidneys, and increase the half-life period [31]. Another important feature of P = S modification is that it retains the ability to activate RNase H, thereby recognizing P = S modified DNA-RNA hybrid chains and degrading RNA [32, 33]. However, it is difficult for the first-generation oligonucleotide-modified ASO to pass through the blood-brain barrier, entering the central nervous system [34, 35].

### 3.2. Glycosyl Modification

The glycosyl-modified ASO is called the second-generation ASO. The most important feature is that the cholesterol at the 2′position on the glycosyl is replaced by other high-stability groups and was usually used in combination with P = S in application. The second-generation ASO has three advantages: firstly, the 2′modification blocks the nucleophilic properties of the original 2′hydroxyl group and further increases the resistance of ASO to nucleases; secondly, this modification improves the thermal stability of complementary hybridization, and the increased specificity of binding makes it possible to use shorter oligonucleotide; and thirdly and finally, it can reduce the cytotoxicity caused by P = S modification [36–38]. The most common glycosyl modifications are 2′-O-methyl (OME), 2′-O-methoxyethyl (MOE), and 2′fluoro (2′F), while the nusinersen approved by FDA is using MOE and P = S. It is worth mentioning that the 2′-F ASO itself can recruit the splicing inhibitor ILF2/3 to inhibit splicing [39]. Other glycosyl modifications, such as locked nucleic acids (LNA), 2′,4′-constrained ethyl (cEt), and tricyclo-DNA (tc-DNA), have also been applied to treat neurodegenerative diseases.

### 3.3. Other Modified ASOs

The third-generation ASO combines phosphate, ribose, and nucleoside modifications, which mainly includes peptide nucleic acids (PNA) and phosphorodiamidate morpholine oligomer (PMO). In brief, PNA modification is the connection of chemically modified oligonucleotides to short peptides, where the oligonucleotides perform base complementary pairing to provide targeting, and the short peptides provide functionality such as splicing regulation or translation inhibition. PNA is uncharged, so it also has the advantages of stability and binding specificity and cannot activate RNase H [40, 41]. However, PNA has an obvious disadvantage, that is, its hydrophobicity causes poor cell absorption. However, PNA modified with peptide conjugates can also improve absorption and water solubility. Studies have reported that unmodified PNA can be taken up by neuronal cells in vivo, but the application of PNA antisense oligonucleotides in neurodegenerative diseases is still limited [42, 43]. When administered peripherally, PNA will be cleared quickly, which may be the reason why they have not been widely used in the body so far [44]. PMO has a morpholine ring instead of a ribose ring. Similar to PNA, the PMO backbone is neutrally charged, is not compatible with RNase H, and is highly resistant to degradation by nucleases and proteases. Scientists have used the intracerebral injection of PMO-modified ASO to successfully repair splicing, and the PMO-modified ASO for the treatment of DMD, eteplirsen, has also been approved by the FDA [45].

## 4. Delivery Method of Antisense Nucleic Acid Drugs

In neurodegenerative diseases, the destruction of the blood-brain barrier is common. It has been shown in animal models that the damaged blood-brain barrier itself can cause neurodegeneration [46, 47]. Although chemical modification has improved the properties and mechanism of antisense nucleic acid drugs, the currently approved antisense oligonucleotide drugs still cannot cross the blood-brain barrier. Therefore, the application of ASO in the treatment of neurodegenerative diseases is still challenging.

### 4.1. Systemic Administration

For systemic administration, ASO must be selectively transported at an appropriate concentration to cells of the brain and spinal cord tissue through the blood circulation. The general way for small molecules to pass through the blood vessel barrier is simple diffusion. Although chemical modification gives ASOs the ability to bind to hemoglobin, the molecular weight of antisense nucleic acid is relatively large that it cannot reach an effective concentration in the nervous system by simple diffusion through the blood vessel barrier. Early studies have shown that only about one percent of peripherally injected ASOs can be detected in the brain [48, 49]. ASOs cross the vascular barrier through two mechanisms, one of which is receptor-mediated endocytosis. According to the affinity and tight binding properties of streptavidin and biotinase, Lee and colleagues used biotinase to label antisense oligonucleotides, combining streptavidin with a radiolabeled mouse transferrin receptor monoclonal antibody. The tracing results showed that the antisense oligonucleotides combined with the transferrin receptor monoclonal antibody to form a conjugate, which reached the brain through receptor-mediated endocytosis in the transgenic mouse model [50]. The second mechanism is a cell-penetrating peptide- (CPP-) based delivery system. CPP is a positively charged polypeptide chain with a length of about 5–30 amino acids, which transports various macromolecules through the cell membrane [51]. In mice, ASOs transported by an arginine-rich CPP-labeled system were able to cross the blood-brain barrier and were then widely distributed in the mouse brain [52]. However, for ASOs having different chemical properties (with modification), not all ASOs are suitable for CPP coupling, which determines the most effective delivery route to a large extent. In the DMD mouse model, Goyenvalle et al. found that ASOs with tc-DNA structure type can reach the brain tissue and exert the drug effect with peripheral administration [53].

### 4.2. Central Nervous System Drug Delivery

Most ASOs currently used to treat neurodegenerative diseases must be administered by intraventricular injection or intrathecal injection. In these ways, it is unnecessary for ASOs to pass through the blood-brain barrier and can be transported to the cerebrospinal fluid, thereby distributing throughout the central nervous system. This method of administration has certain advantages over peripheral administration. Because of the material exchange between the cerebrospinal fluid and the brain parenchyma, the drug can be directly and quickly delivered to the nervous system and produce a higher drug concentration, which means that a smaller drug dose can be used to achieve the therapeutic effect and minimize the toxicity. Over time, ASOs gradually shift from the cerebrospinal fluid to the blood circulation and can also enter the peripheral tissues. Central nervous system administration of antisense oligonucleotides has been widely used in rodent models and nonhuman primates of neurodegenerative diseases [54]. In clinical trials of ALS and SMA, no side effects of nucleic acid drugs were observed using an intrathecal injection of antisense oligonucleotides and the intrathecal injection of nusinersen has been approved by the FDA [55].

### 4.3. Other Ways of Administration

Nasal administration is an emerging mode of administration. After administration, the drug can enter the nerve center through the olfactory nerve route and the olfactory mucosal epithelial route. Although a few studies have shown that antisense oligonucleotides can be delivered to the brain by intranasal administration so far, this is still a very promising alternative delivery route. Clinical trials have shown that nasal administration of insulin can significantly improve the cognitive ability of patients with diabetes and AD [56]. In addition, cell-penetrating peptide coupled with ethylene glycol polycaprolactone copolymer can accurately deliver siRNA to the brain through nasal administration. In rats, intranasal delivery of oligonucleotide GRN163 can delay the growth of tumors in the brain and prolong survival [57, 58]. Therefore, these studies indicate that intranasal delivery may become an important choice for antisense oligonucleotide delivery in the future.

## 5. Development of Antisense Nucleic Acids in Several Neurodegenerative Diseases

### 5.1. Spinal Muscular Atrophy (SMA)

SMA is a common autosomal recessive genetic disease. It is caused by survival of motor neuron 1(*SMN*1) gene mutation or deletion which will lead to the lack of SMN protein. Its pathological characteristics are degeneration of motor neurons in the anterior horn of the spinal cord, neuromuscular junction necrosis, while the clinical manifestations are of myasthenia and amyotrophy. The parallel homologous gene of gene *SMN*1 in humans, which is called *SMN*2, can express a small amount of SMN protein. *SMN*2 gene is very similar to *SMN*1 gene, with only a few bases different. One of the key base differences is that the sixth base in exon 7 of SMN1 is C, while it is T in SMN2 gene. This does not affect protein coding, but it will severely affect RNA splicing. The result is that about 90% of exon 7 of the SMN2 gene is skipped, while only about 5% of SMN1 is skipped. Therefore, *SMN*2 gene can only produce about 10–20% of the functional *SMN* protein, which is insufficient to compensate for the decrease in *SMN* protein expression caused by *SMN*1 mutations or deletion. Therefore, activating *SMN*2 expression can be used as an important strategy for the treatment of SMA [59–61].

A breakthrough in the use of antisense nucleic acids to treat SMA is that Hua and colleagues working in Cold Spring Harbor used the ASO walk method to systematically and comprehensively screen *SMN*2 exon 7 and flanking introns. They optimized the length and position of ASO by using microwalk, finally found that ASO10-27 can target the intronic splicing silencer-N1 (ISS–N1) element on intron 7 to prevent the binding of the splicing inhibitor HNRNPA1/2, thereby increasing the inclusion of exon 7 MOE P = S modified ASO10-27 (nusinersen once used its name), improved motor function in SMA mouse models, and extended the life span of SMA mice by 25 times [25, 62–64]. Preclinical experiments have shown that intrathecal injection of 3 mg of ASOs into nonhuman primates is well tolerated 24 hours later and is widely distributed in the spinal cord. Finally, in view of the good clinical trial results, the FDA approved nusinersen (Spinraza), the first antisense nucleic acid drug to treat SMA by intrathecal injection into the central nervous system in 2016 [65, 66].

### 5.2. Amyotrophic Lateral Sclerosis (ALS)

ALS is a fatal devastating neurodegenerative disorder which predominantly affects the motor neurons in the brain and spinal cord. The death of motor neurons in ALS causes subsequent muscle atrophy, paralysis, and eventually death. The pathological mechanism of ALS has not been clarified, which is mainly caused by genetic and environmental factors; there is no effective treatment method yet [67, 68]. According to epidemiological investigations, about 10% of ALS cases are hereditary. Altogether 20 genes are linked to familial ALS, most of which are related to 4 genes: mutations in chromosome 9 open reading frame 72 (C9ORF72) which account for about 35% of hereditary ALS, superoxide dismutase 1 (SOD1) mutations which cover about 20%, RNA binding protein FUS/TLS (FUS) mutations which occupy about (1–5%), and TAR DNA binding protein 43 (TDP) -43) taking up approximately (1–5%) [69, 70].

Antisense nucleic acid drugs for the treatment of ALS mainly target the two genes C9ORF72 and SOD1. It is not completely clear how SOD1 gene mutation causes ALS. It may be due to the aggregation of the mutant protein that causes proteins to become toxic. Intrathecal administration of 2′-MOE P = S gapmer ASO targeting SOD1 mutant mRNA significantly reduces the levels of SOD1 protein, mRNA in the brain and spinal cord of rats. Preonset administration can delay disease progression and prolong the survival period [71]. The first phase of clinical trials studied the safety of different doses of ASO when injected into the cerebrospinal fluid of ALS patients. The results showed that the drug did not have serious side effects [72]. There are GGGGCC (G4C2) duplications in intron 1 of the C9ORF72 gene. Normal people have about 2–20 duplications with few being able to reach 30, which in ALS patients; however, they can reach 700–1600 or even several thousand. In this case, people with hundreds of G4C2 duplications can be diagnosed as patients in clinic [73, 74]. It is not clear how the increased number of G4C2 repeats of the C9ORF72 gene causes ALS. DeJesus-Hernandez et al. found that the increase of repeated sequences will generate RNA foci in the nucleus, forming RNA toxic damage to the motor neurons [73]. Donnelly et al. induced differentiation of C9orf72-positive pluripotent stem cells into motor neuron cells and found that RNA foci were expressed in the nucleus [75]. The researchers used 2′-MOE P = S gapmer ASO to target exon 2 shared by the c9orf72 transcript and the intron sequence near the repetitive sequence to degrade target mRNA, thereby reducing pathological RNA foci of fibroblasts and inducing pluripotent stem cells from C9ORF72 ALS patient [75, 76]. The gapmer ASO targeting the C9ORF72 gene has not yet entered the clinical stage.

### 5.3. Duchenne Muscular Dystrophy (DMD)

DMD is a rare and incurable disease due to mutations in the DMD gene located at the Xp21 locus. DMD gene mutation is the main reason for the pathology and progression of neuromuscular diseases that cause neuroatrophy. Most of the mutations found in DMD genes are deletions/duplications and are nonrandomly distributed. Due to the lack of dystrophin, there is progressive muscle weakness, which usually leads to a decrease in muscle membrane elasticity in their twenties and eventually death due to respiratory and heart failure [77–81].

The DMD gene is one of the largest genes in the human genome, and many mutations in this gene have been reported in DMD patients. The latest research showed that, in patients with DMD, 69% have large deletions, 11% have large duplications, 10% have nonsense mutations, 7% have missense mutations, and 3% have introns or other mutations [82]. Some researchers have classified mutations and put forward the hypothesis of reading frame retention: mutations that do not change the reading frame may retain part of the protein function, leading to a milder phenotype of Becker muscular dystrophy (BMD). Meanwhile, mutations that disrupt the reading frame are more likely to cause a severe DMD phenotype [83]. This hypothesis was confirmed to be consistent with 91% of DMD patients [84]. Based on this, inducing exon skipping with frameshift mutations to correct the reading frame has become an important strategy for the treatment of DMD. It has been reported that the strategy of correcting reading frames using exon skipping may be applicable to approximately 83% of DMD patients, of which approximately 13% of DMD patients can be relieved by exon 51 skipping [85].

Two ASOs with splicing regulation effects modified by 2′oMeP = S (drisapersen) and PMO (eteplirsen) have been successfully applied to induce exon 51 skipping. Yokota et al. found that intramuscular injection of drisapersen and eteplirsen in a dog model of muscular dystrophy can effectively increase exon skipping, inducing dystrophin expression and thus improving its clinical phenotype [86, 87]. Drisapersen performed well in the first phase of clinical trials, but the results of the second phase of clinical trials showed that the improvement effect of drugs treated by subcutaneous administration was not obvious, and adverse reactions such as proteinuria occurred, which finally resulted in the termination of the drug development with great regret [88, 89]. Eteplirsen, which was injected intravenously, was well tolerated in clinical trials and was approved by the FDA in September 2016 [90, 91]. Although it has been approved, eteplirsen is poorly absorbed in muscle tissue and cannot be stable in the body for a long time, so it needs to be injected for life [92].

## 6. Conclusions

The latest progress of antisense oligonucleotides in the treatment of neurodegenerative diseases is encouraging. This series of progress is the result of joint efforts of chemists, biologists, and clinicians driven by the lack of effective drugs for decades. Antisense nucleic acid technology is actually a targeted therapy, which relies on clear pathogenesis. The promotion of this technology will surely promote research in basic medicine. The success of Nusinersen et al. confirmed the broad prospects of ASOs in the treatment of neurodegenerative diseases, which also paved the way for the use of ASO strategies to treat a wider range of diseases with known pathogenesis. It is believed that this technology not only has the potential to overcome more neurological diseases in the near future but also shines in other diseases such as cancer.

## Data Availability

No data were used to support this paper.

## References

[1] Moretti M., Fraga D. B., Rodrigues A. L. S. (2017). Preventive and therapeutic potential of ascorbic acid in neurodegenerative diseases. *CNS Neuroscience & Therapeutics*.

[2] Paillusson S., Stoica R., Gomez-Suaga P. (2016). There’s something wrong with my MAM; the ER-mitochondria axis and neurodegenerative diseases. *Trends in Neurosciences*.

[3] Wyant K. J., Ridder A. J., Dayalu P. (2017). Huntington’s disease-update on treatments. *Current Neurology and Neuroscience Reports*.

[4] Lang Y., Chu F., Shen D. (2018). Role of inflammasomes in neuroimmune and neurodegenerative diseases: a systematic review. *Mediators of Inflammation*.

[5] Dohm C. P., Kermer P., Bahr M. (2008). Aggregopathy in neurodegenerative diseases: mechanisms and therapeutic implication. *Neurodegenerative Diseases*.

[6] Rotermund C., Machetanz G., Fitzgerald J. C. (2018). The therapeutic potential of metformin in neurodegenerative diseases. *Frontiers in Endocrinology (Lausanne)*.

[7] Gandhi S., Wood N. W. (2005). Molecular pathogenesis of Parkinson’s disease. *Human Molecular Genetics*.

[8] Socias S. B., Gonzalez-Lizarraga F., Avila C. L. (2018). Exploiting the therapeutic potential of ready-to-use drugs: repurposing antibiotics against amyloid aggregation in neurodegenerative diseases. *Progress in Neurobiology*.

[9] Soto C., Estrada L. D. (2008). Protein misfolding and neurodegeneration. *Archives in Neurology*.

[10] Skaper S. D. (2007). The brain as a target for inflammatory processes and neuroprotective strategies. *Annals of New York Academy of Sciences*.

[11] Rinaldi C., Wood M. J. A. (2018). Antisense oligonucleotides: the next frontier for treatment of neurological disorders. *Nature Reviews Neurology*.

[12] Zamecnik P. C., Stephenson M. L. (1978). Inhibition of rous sarcoma virus replication and cell transformation by a specific oligodeoxynucleotide. *Proceedings of the National Academy of Sciences of the United States of America*.

[13] Geary R. S., Henry S. P., Grillone L. R. (2002). Fomivirsen: clinical pharmacology and potential drug interactions. *Clinical Pharmacokinetics*.

[14] Vickers T. A., Crooke S. T. (2015). The rates of the major steps in the molecular mechanism of RNase H1-dependent antisense oligonucleotide induced degradation of RNA. *Nucleic Acids Research*.

[15] Lennox K. A., Behlke M. A. (2016). Cellular localization of long non-coding RNAs affects silencing by RNAi more than by antisense oligonucleotides. *Nucleic Acids Research*.

[16] Monia B. P., Lesnik E. A., Gonzalez C. (1993). Evaluation of 2′-modified oligonucleotides containing 2′-deoxy gaps as antisense inhibitors of gene expression. *Journal of Biological Chemistry*.

[17] Minshull J., Hunt T. (1986). The use of single-stranded DNA and RNase H to promote quantitative “hybrid arrest of translation” of mRNA/DNA hybrids in reticulocyte lysate cell-free translations. *Nucleic Acids Research*.

[18] Nakamura H., Oda Y., Iwai S. (1991). How does RNase H recognize a DNA.RNA hybrid?. *Proceedings of the National Academy of Sciences of the United States of America*.

[19] Hu J., Matsui M., Gagnon K. T. (2009). Allele-specific silencing of mutant huntingtin and ataxin-3 genes by targeting expanded CAG repeats in mRNAs. *Nature Biotechnology*.

[20] Chauhan N. B., Siegel G. J. (2007). Antisense inhibition at the beta-secretase-site of beta-amyloid precursor protein reduces cerebral amyloid and acetyl cholinesterase activity in Tg2576. *Neuroscience*.

[21] Kelemen O., Convertini P., Zhang Z. (2013). Function of alternative splicing. *Gene*.

[22] Keren H., Lev-Maor G., Ast G. (2010). Alternative splicing and evolution: diversification, exon definition and function. *Nature Reviews Genetics*.

[23] Havens M. A., Hastings M. L. (2016). Splice-switching antisense oligonucleotides as therapeutic drugs. *Nucleic Acids Research*.

[24] Zalachoras I., Grootaers G., van Weert L. T. (2013). Antisense-mediated isoform switching of steroid receptor coactivator-1 in the central nucleus of the amygdala of the mouse brain. *BMC Neuroscience*.

[25] Singh N. K., Singh N. N., Androphy E. J., Singh R. N. (2006). Splicing of a critical exon of human survival motor neuron is regulated by a unique silencer element located in the last intron. *Molecular and Cellular Biology*.

[26] Evers M. M., Tran H. D., Zalachoras I. (2013). Ataxin-3 protein modification as a treatment strategy for spinocerebellar ataxia type 3: removal of the CAG containing exon. *Neurobiology of Disease*.

[27] Du L., Pollard J. M., Gatti R. A. (2007). Correction of prototypic ATM splicing mutations and aberrant ATM function with antisense morpholino oligonucleotides. *Proceedings of the National Academy of Sciences of the United States of America*.

[28] Eder P. S., DeVine R. J., Dagle J. M., Walder J. A. (1991). Substrate specificity and kinetics of degradation of antisense oligonucleotides by a 3′ exonuclease in plasma. *Antisense Research and Development*.

[29] Yokota T., Takeda S., Lu Q. L., Partridge T. A., Nakamura A., Hoffman E. P. (2009). A renaissance for antisense oligonucleotide drugs in neurology: exon skipping breaks new ground. *Archives of Neurology*.

[30] Ogawa S., Brown H. E., Okano H. J., Pfaff D. W. (1995). Cellular uptake of intracerebrally administered oligodeoxynucleotides in mouse brain. *Regulatory Peptides*.

[31] Dowdy S. F. (2017). Overcoming cellular barriers for RNA therapeutics. *Nature Biotechnology*.

[32] Wu H., Lima W. F., Zhang H., Fan A., Sun H., Crooke S. T. (2004). Determination of the role of the human RNase H1 in the pharmacology of DNA-like antisense drugs. *Journal of Biological Chemistry*.

[33] Bennett C. F., Swayze E. E. (2010). RNA targeting therapeutics: molecular mechanisms of antisense oligonucleotides as a therapeutic platform. *Annual Review of Pharmacology and Toxicology*.

[34] Phillips J. A., Craig S. J., Bayley D., Christian R. A., Geary R., Nicklin P. L. (1997). Pharmacokinetics, metabolism, and elimination of a 20-mer phosphorothioate oligodeoxynucleotide (CGP 69846A) after intravenous and subcutaneous administration. *Biochemical Pharmacology*.

[35] Geary R. S., Norris D., Yu R., Bennett C. F. (2015). Pharmacokinetics, biodistribution and cell uptake of antisense oligonucleotides. *Advanced Drug Delivery Reviews*.

[36] Zhao Q., Temsamani J., Iadarola P. L., Jiang Z., Agrawal S. (1996). Effect of different chemically modified oligodeoxynucleotides on immune stimulation. *Biochemical Pharmacology*.

[37] Freier S. M., Altmann K. H. (1997). The ups and downs of nucleic acid duplex stability: structure-stability studies on chemically-modified DNA:RNA duplexes. *Nucleic Acids Research*.

[38] Geary R. S., Watanabe T. A., Truong L. (2001). Pharmacokinetic properties of 2′-O-(2-methoxyethyl)-modified oligonucleotide analogs in rats. *Journal of Pharmacology and Experimental Therapeutics*.

[39] Rigo F., Hua Y., Chun S. J., Prakash T. P., Krainer A. R., Bennett C. F. (2012). Synthetic oligonucleotides recruit ILF2/3 to RNA transcripts to modulate splicing. *Nature Chemical Biology*.

[40] Egholm M., Buchardt O., Christensen L. (1993). PNA hybridizes to complementary oligonucleotides obeying the Watson-Crick hydrogen-bonding rules. *Nature*.

[41] Demidov V. V., Potaman V. N., Frank-Kamenetskii M. D. (1994). Stability of peptide nucleic acids in human serum and cellular extracts. *Biochemical Pharmacology*.

[42] Turner Y., Wallukat G., Saalik P., Wiesner B., Pritz S., Oehlke J. (2010). Cellular uptake and biological activity of peptide nucleic acids conjugated with peptides with and without cell-penetrating ability. *Journal of Peptide Science*.

[43] Tyler B. M., McCormick D. J., Hoshall C. V. (1998). Specific gene blockade shows that peptide nucleic acids readily enter neuronal cells in vivo. *FEBS Letters*.

[44] McMahon B. M., Mays D., Lipsky J., Stewart J. A., Fauq A., Richelson E. (2002). Pharmacokinetics and tissue distribution of a peptide nucleic acid after intravenous administration. *Antisense and Nucleic Acid Drug Development*.

[45] Wu B., Moulton H. M., Iversen P. L. (2008). Effective rescue of dystrophin improves cardiac function in dystrophin-deficient mice by a modified morpholino oligomer. *Proceedings of the National Academy of Sciences of the United States of America*.

[46] Tomkins O., Friedman O., Ivens S. (2007). Blood-brain barrier disruption results in delayed functional and structural alterations in the rat neocortex. *Neurobiology of Disease*.

[47] Winkler E. A., Sengillo J. D., Sagare A. P. (2014). Blood-spinal cord barrier disruption contributes to early motor-neuron degeneration in ALS-model mice. *Proceedings of the National Academy of Sciences of the United States of America*.

[48] Banks W. A., Farr S. A., Butt W., Kumar V. B., Franko M. W., Morley J. E. (2001). Delivery across the blood-brain barrier of antisense directed against amyloid beta: reversal of learning and memory deficits in mice overexpressing amyloid precursor protein. *Journal of Pharmacology and Experimental Therapeutics*.

[49] Khorkova O., Wahlestedt C. (2017). Oligonucleotide therapies for disorders of the nervous system. *Nature Biotechnology*.

[50] Lee H. J., Boado R. J., Braasch D. A., Corey D. R., Pardridge W. M. (2002). Imaging gene expression in the brain in vivo in a transgenic mouse model of Huntington’s disease with an antisense radiopharmaceutical and drug-targeting technology. *The Journal of Nuclear Medicine*.

[51] Lehto T., Kurrikoff K., Langel U. (2012). Cell-penetrating peptides for the delivery of nucleic acids. *Expert Opinion on Drug Delivery*.

[52] Du L., Kayali R., Bertoni C. (2011). Arginine-rich cell-penetrating peptide dramatically enhances AMO-mediated ATM aberrant splicing correction and enables delivery to brain and cerebellum. *Human Molecular Genetics*.

[53] Goyenvalle A., Griffith G., Babbs A. (2015). Functional correction in mouse models of muscular dystrophy using exon-skipping tricyclo-DNA oligomers. *Nature Medicine*.

[54] Kordasiewicz H. B., Stanek L. M., Wancewicz E. V. (2012). Sustained therapeutic reversal of Huntington’s disease by transient repression of huntingtin synthesis. *Neuron*.

[55] Finkel R. S., Mercuri E., Darras B. T. (2017). Nusinersen versus sham control in infantile-onset spinal muscular atrophy. *The New England Journal of Medicine*.

[56] Claxton A., Baker L. D., Wilkinson C. W. (2013). Sex and ApoE genotype differences in treatment response to two doses of intranasal insulin in adults with mild cognitive impairment or Alzheimer’s disease. *Journal of Alzheimer’s Disease*.

[57] Kanazawa T., Akiyama F., Kakizaki S., Takashima Y., Seta Y. (2013). Delivery of siRNA to the brain using a combination of nose-to-brain delivery and cell-penetrating peptide-modified nano-micelles. *Biomaterials*.

[58] Hashizume R., Ozawa T., Gryaznov S. M. (2008). New therapeutic approach for brain tumors: intranasal delivery of telomerase inhibitor GRN163. *Neuro-Oncology*.

[59] Prior T. W., Leach M. E., Finanger E., Adam M. P., Ardinger H. H., Pagon R. A. (1993). Spinal muscular atrophy. *GeneReviews*.

[60] Brzustowicz L. M., Lehner T., Castilla L. H. (1990). Genetic mapping of chronic childhood-onset spinal muscular atrophy to chromosome 5q11.2-13.3. *Nature*.

[61] Burghes A. H., Beattie C. E. (2009). Spinal muscular atrophy: why do low levels of survival motor neuron protein make motor neurons sick?. *Nature Reviews Neuroscience*.

[62] Hua Y., Vickers T. A., Okunola H. L., Bennett C. F., Krainer A. R. (2008). Antisense masking of an hnRNP A1/A2 intronic splicing silencer corrects SMN2 splicing in transgenic mice. *American Journal of Human Genetics*.

[63] Hua Y., Sahashi K., Rigo F. (2011). Peripheral SMN restoration is essential for long-term rescue of a severe spinal muscular atrophy mouse model. *Nature*.

[64] Hua Y., Vickers T. A., Baker B. F., Bennett C. F., Krainer A. R. (2007). Enhancement of SMN2 exon 7 inclusion by antisense oligonucleotides targeting the exon. *PLoS Biology*.

[65] Hache M., Swoboda K. J., Sethna N. (2016). Intrathecal injections in children with spinal muscular atrophy: nusinersen clinical trial experience. *Journal of Child Neurology*.

[66] Aartsma-Rus A. (2017). FDA approval of nusinersen for spinal muscular atrophy makes 2016 the year of splice modulating oligonucleotides. *Nucleic Acid Therapeutics*.

[67] Wingo T. S., Cutler D. J., Yarab N., Kelly C. M., Glass J. D. (2011). The heritability of amyotrophic lateral sclerosis in a clinically ascertained United States research registry. *PLoS One*.

[68] Al-Chalabi A., Hardiman O. (2013). The epidemiology of ALS: a conspiracy of genes, environment and time. *Nature Reviews Neurology*.

[69] Corcia P., Couratier P., Blasco H. (2017). Genetics of amyotrophic lateral sclerosis. *Revue Neurologique (Paris)*.

[70] van Es M. A., Hardiman O., Chio A. (2017). Amyotrophic lateral sclerosis. *The Lancet*.

[71] Smith R. A., Miller T. M., Yamanaka K. (2006). Antisense oligonucleotide therapy for neurodegenerative disease. *Jouranal of Clinical Investigation*.

[72] Miller T. M., Pestronk A., David W. (2013). An antisense oligonucleotide against SOD1 delivered intrathecally for patients with SOD1 familial amyotrophic lateral sclerosis: a phase 1, randomised, first-in-man study. *The Lancet Neurology*.

[73] DeJesus-Hernandez M., Mackenzie I. R., Boeve B. F. (2011). Expanded GGGGCC hexanucleotide repeat in noncoding region of C9ORF72 causes chromosome 9p-linked FTD and ALS. *Neuron*.

[74] Wood H. (2011). A hexanucleotide repeat expansion in C9ORF72 links amyotrophic lateral sclerosis and frontotemporal dementia. *Nature Reviews Neurology*.

[75] Donnelly C. J., Zhang P. W., Pham J. T. (2013). RNA toxicity from the ALS/FTD C9ORF72 expansion is mitigated by antisense intervention. *Neuron*.

[76] Lagier-Tourenne C., Baughn M., Rigo F. (2013). Targeted degradation of sense and antisense C9orf72 RNA foci as therapy for ALS and frontotemporal degeneration. *Proceedings of the National Academy of Sciences of the United States of America*.

[77] Muntoni F., Torelli S., Ferlini A. (2003). Dystrophin and mutations: one gene, several proteins, multiple phenotypes. *The Lancet Neurology*.

[78] Bushby K., Finkel R., Birnkrant D. J. (2010). Diagnosis and management of Duchenne muscular dystrophy, part 1: diagnosis, and pharmacological and psychosocial management. *The Lancet Neurology*.

[79] Lapidos K. A., Kakkar R., McNally E. M. (2004). The dystrophin glycoprotein complex: signaling strength and integrity for the sarcolemma. *Circulation Research*.

[80] Serasinghe M. N., Chipuk J. E. (2017). Mitochondrial fission in human diseases. *Handbook of Expermental Pharmacology*.

[81] Bertero E., Maack C. (2018). Calcium signaling and reactive oxygen species in mitochondria. *Circulation Research*.

[82] Bladen C. L., Salgado D., Monges S. (2015). The TREAT-NMD DMD global database: analysis of more than 7,000 Duchenne muscular dystrophy mutations. *Human Mutation*.

[83] Monaco A. P., Bertelson C. J., Liechti-Gallati S., Moser H., Kunkel L. M. (1988). An explanation for the phenotypic differences between patients bearing partial deletions of the DMD locus. *Genomics*.

[84] Aartsma-Rus A., Van Deutekom J. C., Fokkema I. F., Van Ommen G. J., Den Dunnen J. T. (2006). Entries in the Leiden Duchenne muscular dystrophy mutation database: an overview of mutation types and paradoxical cases that confirm the reading-frame rule. *Muscle & Nerve*.

[85] Aartsma-Rus A., Fokkema I., Verschuuren J. (2009). Theoretic applicability of antisense-mediated exon skipping for Duchenne muscular dystrophy mutations. *Human Mutation*.

[86] McClorey G., Wood M. J. (2015). An overview of the clinical application of antisense oligonucleotides for RNA-targeting therapies. *Current Opinion Pharmacology*.

[87] Yokota T., Hoffman E., Takeda S. (2011). Antisense oligo-mediated multiple exon skipping in a dog model of duchenne muscular dystrophy. *Methods in Molecular Biology*.

[88] Goemans N. M., Tulinius M., van den Akker J. T. (2011). Systemic administration of PRO051 in Duchenne’s muscular dystrophy. *The New England Journal of Medicine*.

[89] Voit T., Topaloglu H., Straub V. (2014). Safety and efficacy of drisapersen for the treatment of Duchenne muscular dystrophy (DEMAND II): an exploratory, randomised, placebo-controlled phase 2 study. *The Lancet Neurology*.

[90] Mendell J. R., Goemans N., Lowes L. P. (2016). Longitudinal effect of eteplirsen versus historical control on ambulation in Duchenne muscular dystrophy. *Annals of Neurology*.

[91] Dowling J. J. (2016). Eteplirsen therapy for Duchenne muscular dystrophy: skipping to the front of the line. *Nature Reviews Neurology*.

[92] Lim K. R., Maruyama R., Yokota T. (2017). Eteplirsen in the treatment of Duchenne muscular dystrophy. *Drug Design, Development and Therapy*.

